# Bis(acetyl­acetonato-κ^2^ *O*,*O*′)(pyridine-κ*N*)zinc(II)

**DOI:** 10.1107/S1600536811019854

**Published:** 2011-05-28

**Authors:** Sanjaya Brahma, M. Srinidhi, S. A. Shivashankar, T. Narasimhamurthy, R. S. Rathore

**Affiliations:** a Materials Research Center, Indian Institute of Science, Bangalore 560 012, India; b Solid State Structural Chemistry Unit, Indian Institute of Science, Bangalore, 560 012, India; c Bioinformatics Infrastructure Facility, School of Life Science, University of Hyderabad, Hyderabad 500 046, India

## Abstract

In the title compound, [Zn(C_5_H_7_O_2_)_2_(C_5_H_5_N)], the metal atom has square-pyramidal coordination geometry with the basal plane defined by the four O atoms of the chelating acetyl­acetonate ligands and with the axial position occupied by the pyridine N atom. The crystal packing is characterized by a C—H⋯O hydrogen-bonded ribbon structure approximately parallel to [10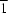].

## Related literature

For related structures, see: Brahma *et al.* (2008 ▶); Neelgund *et al.* (2007 ▶); Urs *et al.* (2001 ▶).
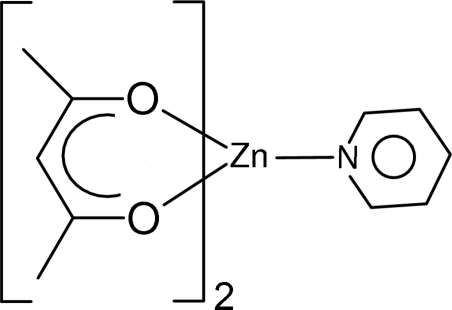


## Experimental

### 

#### Crystal data

[Zn(C_5_H_7_O_2_)_2_(C_5_H_5_N)]
*M* *_r_* = 342.68
Monoclinic, 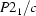

*a* = 7.846 (5) Å
*b* = 27.047 (4) Å
*c* = 8.199 (5) Å
β = 117.984 (3)°
*V* = 1536.5 (14) Å^3^
*Z* = 4
Mo *K*α radiation
μ = 1.61 mm^−1^
*T* = 295 K
0.32 × 0.23 × 0.12 mm

#### Data collection

Bruker APEXII CCD area-detector diffractometer
Absorption correction: multi-scan (*SADABS*; Bruker, 2004 ▶) *T*_min_ = 0.64, *T*_max_ = 0.83
10840 measured reflections
2939 independent reflections
2568 reflections with *I* > 2σ(*I*)
*R*_int_ = 0.074

#### Refinement

*R*[*F* ^2^ > 2σ(*F* ^2^)] = 0.040
*wR*(*F* ^2^) = 0.106
*S* = 0.99
2939 reflections
194 parameters
H-atom parameters constrained
Δρ_max_ = 0.36 e Å^−3^
Δρ_min_ = −0.74 e Å^−3^

### 

Data collection: *APEX2* (Bruker, 2004 ▶); cell refinement: *SAINT-Plus* (Bruker, 2004 ▶); data reduction: *SAINT-Plus*; program(s) used to solve structure: *SHELXS97* (Sheldrick, 2008 ▶); program(s) used to refine structure: *SHELXL97* (Sheldrick, 2008 ▶); molecular graphics: *ORTEP-3* (Farrugia, 1997 ▶) and *PLATON* (Spek, 2009 ▶); software used to prepare material for publication: *SHELXL97* and *PLATON*.

## Supplementary Material

Crystal structure: contains datablocks global, I. DOI: 10.1107/S1600536811019854/ng5159sup1.cif

Structure factors: contains datablocks I. DOI: 10.1107/S1600536811019854/ng5159Isup2.hkl

Additional supplementary materials:  crystallographic information; 3D view; checkCIF report

## Figures and Tables

**Table 1:** Hydrogen-bond geometry (Å, °)

*D*—H⋯*A*	*D*—H	H⋯*A*	*D*⋯*A*	*D*—H⋯*A*
C13—H13⋯O2^i^	0.93	2.50	3.141 (5)	126
C14—H14⋯O3^ii^	0.93	2.59	3.500 (5)	165
C4—H4*A*⋯O4^iii^	0.96	2.41	3.304 (5)	155

Symmetry codes: (i) 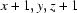; (ii) 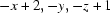; (iii) 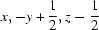.

**Fig. 1.**
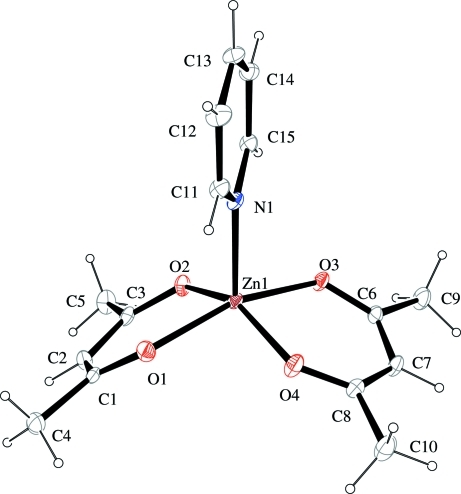
A view of (I) with non-H atoms shown as probability ellipsoids at 30% levels.

**Fig. 2.**
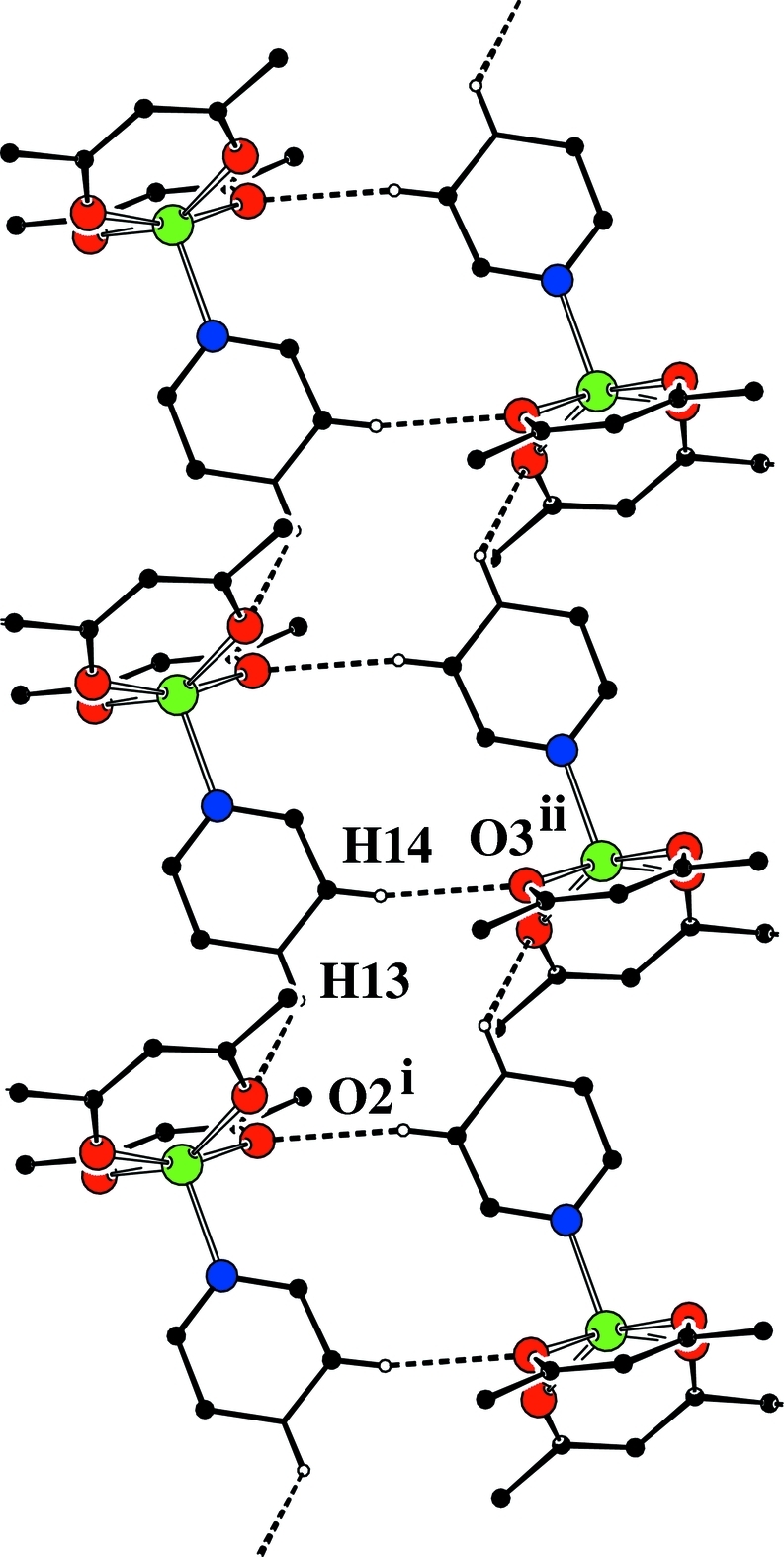
C—H···O hydrogen bonded ribbon structure in (I)

## supplementary crystallographic information

### Comment

The title compound, [Zn(II)(C_5_H_7_O_2_)_2_(C_5_H_5_N)], is a mixed-ligand metal-organic precursor for chemical vapour deposition, with the Zn atom being five coordinate. Metal-organic (MO) complexes have been widely employed as precursors for chemical vapour deposition (CVD) for the growth of various thin films. The title complex, (I), has been synthesized and discussed here. Several such MOCVD precursors have been previously synthesized and characterized (Urs *et al.*, 2001; Neelgund *et al.*, 2007; Brahma *et al.*, 2008; and references therein).

The structure of (I) with adopted atom-numbering scheme is shown in Fig 1. The coordination geometry around Zn(II) is square-pyramidal with the basal plane defined by four O atoms from two chelating acetylacetonate (acac) ligands and the axial position occupied by N atom from pyridine ring. The five-membered ring formed by acetylacetonate and Zn atom is significantly non-planar.

The geometric parameters for observed short contacts are listed in Table 1. Crystal packing diagram is shown in Fig 2. The intermolecular C13—H13···O2 and C14—H14..O3 interactions, combined together generate C—H···O bonded ribbon structure that is approximately parallel to [101]-direction. A short C4—H4A···O4 contact associated with methyl group is also observed in the crystal.

### Experimental

The title complex was synthesized from their precursor hydrate complex, *i.e.* bis(acetylacetonato)aquazinc(II). Acetylacetone (10 mmol, 1.02 ml) was added to zinc diacetate dihydrate solution (5 mmol, 1.099 g; 30% ethanol-water mixture). Potassium hydroxide (KOH) solution (10 mmol, 0.56 g; 30% ethanol-water mixture) was added gradually to achieve a pH of 6–7. After stirring at room temperature for 1 hr, the mixture yielded a precipitate, which was filtered off and dried in a vacuum. The product was recrystallized from ethanol, giving a pure hydrate complex. To obtain the title complex from the hydrate, an ethanol solution of the hydrate was prepared and added in a (1:1) molar ratio to ethanol solutions of pyridine and stirred for 12 hr. Single crystals suitable for X-ray diffraction were grown by slow evaporation of the resultant solution in ethanol at low temperature.

### Refinement

The refections (1,0,0) and (1 1 0) were omitted as they were affected by extinction or absorption. Hydrogen atoms were placed in their stereochemically expected positions and refined with the riding options. The distances with hydrogen atoms are: C(aromatic)—H = 0.93 Å, *C*(methyl)—H = 0.96 Å, and *U*_iso_ = 1.2 *U*_eq_(parent) [1.5 *U*_eq_(parent) for methyl groups].

### Crystal data

**Table:**

[Zn(C_5_H_7_O_2_)_2_(C_5_H_5_N)]	*F*(000) = 712
*M**_r_* = 342.68	*D*_x_ = 1.481 Mg m^−^^3^
Monoclinic, *P*2_1_/*c*	Mo *K*α radiation, λ = 0.71073 Å
Hall symbol: -P 2ybc	Cell parameters from 2570 reflections
*a* = 7.846 (5) Å	θ = 1.5–26°
*b* = 27.047 (4) Å	µ = 1.61 mm^−^^1^
*c* = 8.199 (5) Å	*T* = 295 K
β = 117.984 (3)°	Needle, colorless
*V* = 1536.5 (14) Å^3^	0.32 × 0.23 × 0.12 mm
*Z* = 4	

### Data collection

**Table:**

Bruker APEXII CCD area-detector diffractometer	2939 independent reflections
Radiation source: fine-focus sealed tube	2568 reflections with *I* > 2σ(*I*)
graphite	*R*_int_ = 0.074
φ and ω scans	θ_max_ = 26.0°, θ_min_ = 1.5°
Absorption correction: multi-scan (*SADABS*; Bruker, 2004)	*h* = −9→9
*T*_min_ = 0.64, *T*_max_ = 0.83	*k* = −33→33
10840 measured reflections	*l* = −10→10

### Refinement

**Table:**

Refinement on *F*^2^	Primary atom site location: structure-invariant direct methods
Least-squares matrix: full	Secondary atom site location: difference Fourier map
*R*[*F*^2^ > 2σ(*F*^2^)] = 0.040	Hydrogen site location: inferred from neighbouring sites
*wR*(*F*^2^) = 0.106	H-atom parameters constrained
*S* = 0.99	*w* = 1/[σ^2^(*F*_o_^2^) + (0.0548*P*)^2^] where *P* = (*F*_o_^2^ + 2*F*_c_^2^)/3
2939 reflections	(Δ/σ)_max_ = 0.001
194 parameters	Δρ_max_ = 0.36 e Å^−^^3^
0 restraints	Δρ_min_ = −0.74 e Å^−^^3^

### Special details

**Table:**

Geometry. All e.s.d.'s (except the e.s.d. in the dihedral angle between two l.s. planes) are estimated using the full covariance matrix. The cell e.s.d.'s are taken into account individually in the estimation of e.s.d.'s in distances, angles and torsion angles; correlations between e.s.d.'s in cell parameters are only used when they are defined by crystal symmetry. An approximate (isotropic) treatment of cell e.s.d.'s is used for estimating e.s.d.'s involving l.s. planes.
Refinement. Refinement of *F*^2^ against ALL reflections. The weighted *R*-factor *wR* and goodness of fit *S* are based on *F*^2^, conventional *R*-factors *R* are based on *F*, with *F* set to zero for negative *F*^2^. The threshold expression of *F*^2^ > σ(*F*^2^) is used only for calculating *R*-factors(gt) *etc*. and is not relevant to the choice of reflections for refinement. *R*-factors based on *F*^2^ are statistically about twice as large as those based on *F*, and *R*- factors based on ALL data will be even larger.

### Fractional atomic coordinates and isotropic or equivalent isotropic displacement parameters (Å^2^)

**Table:**

	*x*	*y*	*z*	*U*_iso_*/*U*_eq_	
C1	1.0371 (4)	0.17049 (11)	−0.0161 (4)	0.0224 (7)	
C2	0.9678 (5)	0.12881 (11)	−0.1366 (5)	0.0244 (7)	
H2	0.9648	0.1306	−0.2512	0.029*	
C3	0.9046 (4)	0.08575 (11)	−0.0895 (4)	0.0212 (7)	
C4	1.1147 (5)	0.21268 (12)	−0.0821 (5)	0.0320 (8)	
H4A	1.0578	0.2431	−0.0715	0.048*	
H4B	1.0835	0.2074	−0.2088	0.048*	
H4C	1.2524	0.2144	−0.0079	0.048*	
C5	0.8558 (5)	0.04217 (12)	−0.2241 (4)	0.0309 (8)	
H5A	0.9542	0.0173	−0.1707	0.046*	
H5B	0.8492	0.0536	−0.3379	0.046*	
H5C	0.7336	0.0284	−0.2480	0.046*	
C6	0.5739 (4)	0.11064 (12)	0.2966 (4)	0.0208 (6)	
C7	0.5487 (4)	0.16068 (11)	0.3209 (4)	0.0236 (7)	
H7	0.4373	0.1695	0.3271	0.028*	
C8	0.6730 (4)	0.19786 (11)	0.3364 (4)	0.0221 (7)	
C9	0.4272 (4)	0.07458 (12)	0.2835 (5)	0.0298 (8)	
H9A	0.3979	0.0526	0.1817	0.045*	
H9B	0.3123	0.0918	0.2643	0.045*	
H9C	0.4760	0.0558	0.3959	0.045*	
C10	0.6314 (5)	0.25065 (12)	0.3684 (5)	0.0335 (8)	
H10A	0.7281	0.2615	0.4875	0.050*	
H10B	0.5067	0.2523	0.3633	0.050*	
H10C	0.6329	0.2716	0.2745	0.050*	
C11	1.2801 (4)	0.13543 (11)	0.6180 (4)	0.0221 (7)	
H11	1.2780	0.1688	0.5894	0.026*	
C12	1.4307 (4)	0.11783 (13)	0.7803 (4)	0.0285 (7)	
H12	1.5266	0.1393	0.8595	0.034*	
C13	1.4377 (4)	0.06812 (12)	0.8242 (4)	0.0261 (7)	
H13	1.5385	0.0557	0.9322	0.031*	
C14	1.2931 (4)	0.03751 (11)	0.7054 (4)	0.0238 (7)	
H14	1.2927	0.0040	0.7307	0.029*	
C15	1.1475 (4)	0.05834 (11)	0.5460 (4)	0.0198 (6)	
H15	1.0499	0.0376	0.4647	0.024*	
N1	1.1384 (3)	0.10638 (9)	0.5017 (3)	0.0176 (5)	
O1	1.0419 (3)	0.17533 (8)	0.1475 (3)	0.0242 (5)	
O2	0.8871 (3)	0.07874 (8)	0.0621 (3)	0.0217 (5)	
O3	0.7104 (3)	0.09184 (7)	0.2837 (3)	0.0210 (5)	
O4	0.8203 (3)	0.19193 (8)	0.3239 (3)	0.0263 (5)	
Zn1	0.91346 (4)	0.130795 (11)	0.25584 (4)	0.01638 (14)	

### Atomic displacement parameters (Å^2^)

**Table:**

	*U*^11^	*U*^22^	*U*^33^	*U*^12^	*U*^13^	*U*^23^
C1	0.0176 (14)	0.0202 (16)	0.0358 (17)	0.0069 (12)	0.0179 (14)	0.0082 (13)
C2	0.0246 (17)	0.0297 (19)	0.0240 (16)	0.0000 (12)	0.0156 (14)	0.0032 (13)
C3	0.0099 (13)	0.0284 (18)	0.0237 (15)	−0.0003 (12)	0.0066 (12)	−0.0032 (13)
C4	0.0346 (18)	0.0235 (18)	0.049 (2)	0.0021 (14)	0.0294 (17)	0.0071 (15)
C5	0.0325 (18)	0.0310 (19)	0.0335 (18)	−0.0077 (14)	0.0189 (15)	−0.0101 (15)
C6	0.0128 (14)	0.0285 (18)	0.0217 (15)	0.0005 (12)	0.0086 (12)	0.0032 (13)
C7	0.0154 (14)	0.0273 (17)	0.0314 (16)	0.0022 (12)	0.0138 (13)	−0.0016 (13)
C8	0.0194 (15)	0.0228 (17)	0.0247 (15)	0.0033 (12)	0.0109 (13)	−0.0002 (13)
C9	0.0214 (16)	0.0290 (19)	0.045 (2)	−0.0026 (13)	0.0206 (15)	0.0002 (15)
C10	0.0317 (18)	0.0261 (19)	0.050 (2)	0.0026 (14)	0.0256 (17)	−0.0053 (16)
C11	0.0168 (15)	0.0191 (16)	0.0288 (17)	−0.0032 (11)	0.0095 (14)	−0.0016 (12)
C12	0.0177 (16)	0.0283 (18)	0.0298 (17)	−0.0054 (13)	0.0030 (14)	−0.0026 (14)
C13	0.0151 (14)	0.0343 (19)	0.0226 (15)	0.0043 (12)	0.0035 (13)	0.0038 (14)
C14	0.0223 (15)	0.0200 (16)	0.0297 (16)	0.0029 (12)	0.0128 (13)	0.0049 (13)
C15	0.0152 (14)	0.0197 (15)	0.0235 (15)	−0.0041 (11)	0.0082 (12)	−0.0016 (12)
N1	0.0119 (11)	0.0196 (13)	0.0204 (12)	−0.0004 (9)	0.0069 (10)	−0.0020 (10)
O1	0.0262 (11)	0.0171 (11)	0.0339 (12)	−0.0033 (8)	0.0177 (10)	0.0000 (9)
O2	0.0180 (10)	0.0248 (12)	0.0246 (11)	−0.0059 (8)	0.0119 (9)	−0.0050 (9)
O3	0.0141 (10)	0.0197 (11)	0.0314 (11)	−0.0002 (8)	0.0126 (9)	−0.0009 (9)
O4	0.0217 (11)	0.0194 (12)	0.0430 (13)	−0.0010 (9)	0.0193 (10)	−0.0040 (10)
Zn1	0.0114 (2)	0.0171 (2)	0.0196 (2)	0.00060 (11)	0.00634 (16)	0.00057 (13)

### Geometric parameters (Å, °)

**Table:**

C1—O1	1.331 (4)	C9—H9B	0.9600
C1—C2	1.428 (4)	C9—H9C	0.9600
C1—C4	1.508 (4)	C10—H10A	0.9600
C2—C3	1.390 (4)	C10—H10B	0.9600
C2—H2	0.9300	C10—H10C	0.9600
C3—O2	1.327 (3)	C11—N1	1.329 (4)
C3—C5	1.535 (4)	C11—C12	1.385 (4)
C4—H4A	0.9600	C11—H11	0.9300
C4—H4B	0.9600	C12—C13	1.386 (5)
C4—H4C	0.9600	C12—H12	0.9300
C5—H5A	0.9600	C13—C14	1.373 (4)
C5—H5B	0.9600	C13—H13	0.9300
C5—H5C	0.9600	C14—C15	1.389 (4)
C6—O3	1.234 (3)	C14—H14	0.9300
C6—C7	1.396 (4)	C15—N1	1.342 (4)
C6—C9	1.473 (4)	C15—H15	0.9300
C7—C8	1.365 (4)	N1—Zn1	2.068 (2)
C7—H7	0.9300	O1—Zn1	2.024 (2)
C8—O4	1.218 (4)	O2—Zn1	2.059 (2)
C8—C10	1.515 (4)	O3—Zn1	2.011 (2)
C9—H9A	0.9600	O4—Zn1	1.991 (2)
			
O1—C1—C2	126.6 (3)	C8—C10—H10B	109.5
O1—C1—C4	117.5 (3)	H10A—C10—H10B	109.5
C2—C1—C4	115.9 (3)	C8—C10—H10C	109.5
C3—C2—C1	122.6 (3)	H10A—C10—H10C	109.5
C3—C2—H2	118.7	H10B—C10—H10C	109.5
C1—C2—H2	118.7	N1—C11—C12	122.4 (3)
O2—C3—C2	126.0 (3)	N1—C11—H11	118.8
O2—C3—C5	117.6 (3)	C12—C11—H11	118.8
C2—C3—C5	116.4 (3)	C11—C12—C13	119.7 (3)
C1—C4—H4A	109.5	C11—C12—H12	120.2
C1—C4—H4B	109.5	C13—C12—H12	120.2
H4A—C4—H4B	109.5	C14—C13—C12	118.8 (3)
C1—C4—H4C	109.5	C14—C13—H13	120.6
H4A—C4—H4C	109.5	C12—C13—H13	120.6
H4B—C4—H4C	109.5	C13—C14—C15	117.7 (3)
C3—C5—H5A	109.5	C13—C14—H14	121.2
C3—C5—H5B	109.5	C15—C14—H14	121.2
H5A—C5—H5B	109.5	N1—C15—C14	124.3 (3)
C3—C5—H5C	109.5	N1—C15—H15	117.9
H5A—C5—H5C	109.5	C14—C15—H15	117.9
H5B—C5—H5C	109.5	C11—N1—C15	117.2 (3)
O3—C6—C7	126.8 (3)	C11—N1—Zn1	123.7 (2)
O3—C6—C9	113.6 (3)	C15—N1—Zn1	119.05 (19)
C7—C6—C9	119.6 (3)	C1—O1—Zn1	126.83 (19)
C8—C7—C6	125.8 (3)	C3—O2—Zn1	127.44 (19)
C8—C7—H7	117.1	C6—O3—Zn1	124.0 (2)
C6—C7—H7	117.1	C8—O4—Zn1	128.4 (2)
O4—C8—C7	124.1 (3)	O4—Zn1—O3	89.35 (9)
O4—C8—C10	115.4 (3)	O4—Zn1—O1	87.28 (9)
C7—C8—C10	120.5 (3)	O3—Zn1—O1	161.13 (8)
C6—C9—H9A	109.5	O4—Zn1—O2	150.12 (8)
C6—C9—H9B	109.5	O3—Zn1—O2	86.05 (8)
H9A—C9—H9B	109.5	O1—Zn1—O2	87.66 (9)
C6—C9—H9C	109.5	O4—Zn1—N1	104.45 (10)
H9A—C9—H9C	109.5	O3—Zn1—N1	94.65 (10)
H9B—C9—H9C	109.5	O1—Zn1—N1	104.18 (10)
C8—C10—H10A	109.5	O2—Zn1—N1	105.34 (9)
			
O1—C1—C2—C3	−3.7 (5)	C8—O4—Zn1—O3	−11.8 (3)
C4—C1—C2—C3	175.4 (3)	C8—O4—Zn1—O1	149.6 (3)
C1—C2—C3—O2	5.0 (5)	C8—O4—Zn1—O2	69.1 (3)
C1—C2—C3—C5	−173.4 (3)	C8—O4—Zn1—N1	−106.4 (3)
O3—C6—C7—C8	−0.6 (5)	C6—O3—Zn1—O4	13.0 (2)
C9—C6—C7—C8	179.5 (3)	C6—O3—Zn1—O1	−66.6 (4)
C6—C7—C8—O4	2.9 (5)	C6—O3—Zn1—O2	−137.4 (2)
C6—C7—C8—C10	−178.1 (3)	C6—O3—Zn1—N1	117.5 (2)
N1—C11—C12—C13	0.9 (5)	C1—O1—Zn1—O4	−133.7 (2)
C11—C12—C13—C14	−0.5 (5)	C1—O1—Zn1—O3	−53.7 (4)
C12—C13—C14—C15	0.4 (4)	C1—O1—Zn1—O2	16.8 (2)
C13—C14—C15—N1	−0.5 (5)	C1—O1—Zn1—N1	122.1 (2)
C12—C11—N1—C15	−0.9 (4)	C3—O2—Zn1—O4	64.5 (3)
C12—C11—N1—Zn1	−178.9 (2)	C3—O2—Zn1—O3	146.3 (2)
C14—C15—N1—C11	0.8 (4)	C3—O2—Zn1—O1	−15.9 (2)
C14—C15—N1—Zn1	178.8 (2)	C3—O2—Zn1—N1	−119.9 (2)
C2—C1—O1—Zn1	−11.0 (4)	C11—N1—Zn1—O4	−48.2 (2)
C4—C1—O1—Zn1	169.9 (2)	C15—N1—Zn1—O4	133.9 (2)
C2—C3—O2—Zn1	8.4 (4)	C11—N1—Zn1—O3	−138.7 (2)
C5—C3—O2—Zn1	−173.22 (19)	C15—N1—Zn1—O3	43.4 (2)
C7—C6—O3—Zn1	−9.9 (4)	C11—N1—Zn1—O1	42.6 (2)
C9—C6—O3—Zn1	170.01 (19)	C15—N1—Zn1—O1	−135.3 (2)
C7—C8—O4—Zn1	6.3 (5)	C11—N1—Zn1—O2	134.1 (2)
C10—C8—O4—Zn1	−172.8 (2)	C15—N1—Zn1—O2	−43.8 (2)

### Hydrogen-bond geometry (Å, °)

**Table:**

*D*—H···*A*	*D*—H	H···*A*	*D*···*A*	*D*—H···*A*
C13—H13···O2^i^	0.93	2.50	3.141 (5)	126
C14—H14···O3^ii^	0.93	2.59	3.500 (5)	165
C4—H4A···O4^iii^	0.96	2.41	3.304 (5)	155

Symmetry codes: (i) *x*+1, *y*, *z*+1; (ii) −*x*+2, −*y*, −*z*+1; (iii) *x*, −*y*+1/2, *z*−1/2.
